# Colorectal cancer screening using a stool DNA-based *SDC2* methylation test: a multicenter, prospective trial

**DOI:** 10.1186/s12876-021-01759-9

**Published:** 2021-04-15

**Authors:** Chang Woo Kim, Hyunjin Kim, Hyoung Rae Kim, Bong-Hyeon Kye, Hyung Jin Kim, Byung Soh Min, Tae Jeong Oh, Sungwhan An, Suk-Hwan Lee

**Affiliations:** 1 Department of Surgery, Kyung Hee University Hospital at Gangdong, Kyung Hee University School of Medicine, 892 Dongnam-ro, Seoul, Gangdong-gu 05278 Korea; 2 Department of Surgery, Koo Hospital, 141 Gamsambuk-gil, Daegu, Korea; 3 Department of Surgery, Busan Hangun Hospital, 348 Chungnyeol-daero, Dongnae-gu, Busan, Korea; 4 Department of Surgery, St. Vincent’s Hospital, The Catholic University of Korea, 93 Jungbu-daero, Suwon, Korea; 5 Department of Surgery, Eunpyeong St. Mary’s Hospital, The Catholic University of Korea, Tongil-ro, Seoul, 1021 Korea; 6 Department of Surgery, Severance Hospital, Yonsei University College of Medicine, 50-1 Yonsei-ro, Seoul, Korea; 7 Genomictree, Inc, 44-6 Techno 10-ro Yuseong-gu, Daejeon, Korea

**Keywords:** Colorectal cancer, DNA methylation, Syndecan-2, Early detection, Biomarker

## Abstract

**Background:**

Prevention and early detection of colorectal cancer (CRC) is a global priority, with many countries conducting population-based CRC screening programs. Although colonoscopy is the most accurate diagnostic method for early CRC detection, adherence remains low because of its invasiveness and the need for extensive bowel preparation. Non-invasive fecal occult blood tests or fecal immunochemical tests are available; however, their sensitivity is relatively low. Syndecan-2 (*SDC2*) is a stool-based DNA methylation marker used for early detection of CRC. Using the EarlyTect™-Colon Cancer test, the sensitivity and specificity of *SDC2* methylation in stool DNA for detecting CRC were previously demonstrated to be greater than 90%. Therefore, a larger trial to validate its use for CRC screening in asymptomatic populations is now required.

**Methods:**

All participants will collect their stool (at least 20 g) before undergoing screening colonoscopy. The samples will be sent to a central laboratory for analysis. Stool DNA will be isolated using a GT Stool DNA Extraction kit, according to the manufacturer’s protocol. Before performing the methylation test, stool DNA (2 µg per reaction) will be treated with bisulfite, according to manufacturer’s instructions. *SDC2* and *COL2A1* control reactions will be performed in a single tube. The *SDC2* methylation test will be performed using an AB 7500 Fast Real-time PCR system. C_T_ values will be calculated using the 7500 software accompanying the instrument. Results from the EarlyTect™-Colon Cancer test will be compared against those obtained from colonoscopy and any corresponding diagnostic histopathology from clinically significant biopsied or subsequently excised lesions. Based on these results, participants will be divided into three groups: CRC, polyp, and negative. The following clinical data will be recorded for the participants: sex, age, colonoscopy results, and clinical stage (for CRC cases).

**Discussion:**

This trial investigates the clinical performance of a device that allows quantitative detection of a single DNA marker, *SDC2* methylation, in human stool DNA in asymptomatic populations. The results of this trial are expected to be beneficial for CRC screening and may help make colonoscopy a selective procedure used only in populations with a high risk of CRC.

*Trial registration*: This trial (NCT04304131) was registered at ClinicalTrials.gov on March 11, 2020 and is available at https://clinicaltrials.gov/ct2/show/NCT04304131?cond=NCT04304131&draw=2&rank=1.

## Background

Colorectal cancer (CRC) is the second leading cause of cancer-related death worldwide [1]. Studies estimate that more than 250,000 new cases of CRC and approximately 9000 CRC-related deaths will occur in 2020 in South Korea [2]. The mean 5-year survival rate for early-stage CRC can be as high as 90% but can be less than 10% if metastasis occurs [3]. Therefore, prevention and early detection of CRC has emerged as a significant global initiative, and many countries conduct population-based CRC screening programs [4]. Although colonoscopy is the most accurate diagnostic method for early CRC detection, adherence remains very low due to the invasiveness of the procedure and the need for extensive bowel preparation [5, 6]. Sigmoidoscopy without bowel preparation can be used instead of colonoscopy, but it fails to detect abnormalities between the cecum and sigmoid colon. Digital rectal examination is the easiest diagnostic method, but it is only useful for detecting rectal cancer. Although computed tomography (CT) colonography is a non-invasive method that can visualize the proximal colon above the obstructing lesion in extraluminal views, it is not as accurate as colonoscopy [7]. Noninvasive fecal occult blood tests and fecal immunochemical tests (FITs) are also available; however, their sensitivity is relatively low [8]. As an alternative to overcome these limitations, noninvasive, simple, and accurate molecular diagnostic tests using bodily fluids or feces have been gaining attention. These tests may be used for screening in patients reluctant to undergo screening colonoscopies, thereby allowing colonoscopy to be selectively performed in specific cases to detect early-stage CRC.

CRC arises from an accumulation of genetic and epigenetic abnormalities in the genome [9], including DNA methylation, in which methyl (CH_3_) groups are added to the 5′ position of cytosine (C) in the CpG island of a DNA molecule. Aberrant DNA methylation is associated with tumorigenesis of all types of cancer and has been established as one of the most common molecular alterations in CRC [10–12]. Therefore, specific DNA sites that are aberrantly methylated in colorectal neoplasms are well recognized as potential non-invasive molecular biomarkers for early CRC detection.

Regulatory regions in most genes consist of a sequence dense with CpG islands. If methylation occurs at the gene regulatory region, structural changes in chromatin, including in the CpG island, are induced, resulting in gene silencing [13]. Aberrant DNA methylation often occurs prior to genetic alterations. Thus, abnormal DNA methylation of certain genes may occur in progressive cancer, as well as in precancerous lesions (e.g., adenomatous polyps). Therefore, the presence of methylation of specific genes could be used as a biomarker for the diagnosis of early-stage cancer [14]. Targeting cancer-specific methylated DNA for early cancer detection has many advantages, when compared with other known biomarkers [15].

In cancer tissues, abnormally methylated DNA sites are often shed into the blood and circulate in the form of cell-free DNA. In addition, exfoliated cells from the colonic mucosa are frequently present in feces. Because stool specimens from CRC patients usually contain exfoliated tumor cells, stool-derived DNA testing has emerged as a new strategy for the non-invasive detection of CRC and its precursor lesions [16]. We previously identified syndecan-2 (*SDC2*) as a potential stool-based DNA methylation marker for early CRC detection [17, 18]. Moreover, we reported that detecting *SDC2* methylation has greater than 90% sensitivity and specificity, suggesting that this strategy would be an excellent non-invasive screening test for early diagnosis of CRC [19]. Here, we report the protocol of a larger trial to validate the use of *SDC2* methylation detection in stool DNA as a screening method for CRC in asymptomatic populations.

## Methods/design

### Study design

This study is a prospective, multicenter trial to evaluate the clinical performance of a device (EarlyTect™-Colon Cancer, Genomictree, Daejeon, Korea) that allows for the quantitative detection of a single DNA marker, *SDC2* methylation, in human stool DNA from asymptomatic individuals.

Before the study is initiated, the staff will be trained in all aspects of the study, including the assay procedure and instrument use. All eligible participants scheduled for colonoscopy for any reason will be enrolled in this trial. Before initiating bowel preparation for colonoscopy, all participants will collect at least 20 g of their stool using a plastic collection tube (50 mL) that is included in the kit. The following samples will be considered inappropriate for analysis and excluded: diarrhea or loose stool, quantities less than 20 g, samples collected during bowel preparation, and samples submitted over 2 weeks after collection. Samples collected by the participant for the EarlyTect™-Colon Cancer test will be sent to a central laboratory.

Stool DNA will be isolated using a GT Stool DNA Extraction kit (Genomictree, Inc, Daejeon, Korea) according to the following instructions:

1. All stool samples will be weighed and thoroughly homogenized in the preservative buffer using a multiple vortexer (Thermo Fisher Scientific, Waltham, MA, USA). After homogenization, 2–4 g equivalent of each stool sample will be collected and centrifuged at 2,000 rpm for 2 min (HA-1000-3; Hanil Science Medical, Daejeon, Korea).
2. The supernatants will be discarded, and the pellet will be resuspended in 5.0 mL lysis buffer 1 (Genomictree, Inc, Daejeon, Korea) before incubating for 4 min at room temperature.
3. The samples will be centrifuged at 2000 rpm for 2 min, and the supernatant will be discarded before adding 2.0 mL lysis buffer 2 (Genomictree, Inc, Daejeon, Korea) and 30 μL proteinase K (0.4 mg/mL, Sigma-Aldrich, St. Louis, MO, USA). The samples will then be incubated at 70 °C for 10 min.
4. The samples will be centrifuged at 3000 rpm for 5 min, and 0.75 mL supernatant will be added to the MaXtract High-Density tube (Qiagen, Hilden, Germany). Tris-saturated phenol–chloroform-isoamylalcohol (25:24:1 [v/v]; Sigma-Aldrich, St. Louis, MO, USA) will then be added to the samples.
5. The samples will be thoroughly mixed for 1 min and then centrifuged in a microcentrifuge at maximum speed for 10 min. The supernatant (0.5 mL) will be transferred to a new tube, and 1/10 volume of 3 mol/L sodium acetate (pH 5.2; Welgene, Gyeongsan, Korea) and an equal volume of isopropanol (Merck, Darmstadt, Germany) will be added to the sample.
6. Total nucleic acid will then be precipitated by centrifugation at maximum speed for 10 min. The DNA pellet will be washed with 1.0 mL 70% ethanol and dried. DNA concentration will be measured using the Qubit dsDNA BR Assay Kit (Thermo Fisher Scientific, Waltham, MA, USA). A solid phase magnetic bead-based stool DNA extraction method will also be available.

**Fig. 1:**
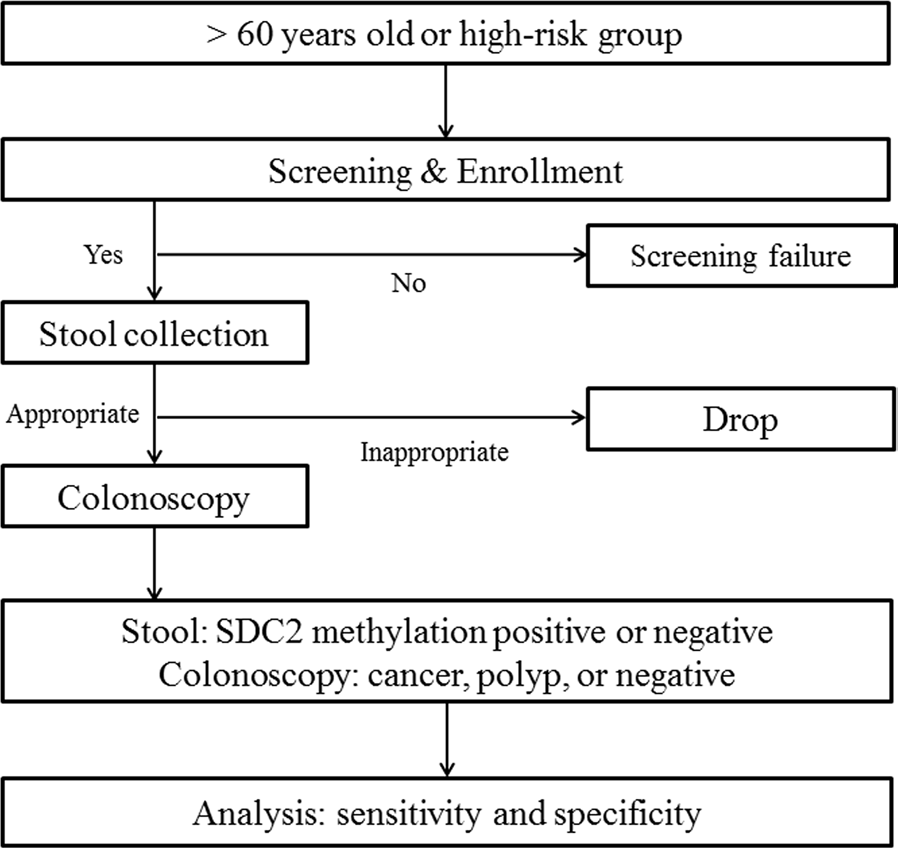
Schematic diagram of the workflow of the trial

All information regarding the stool sample will be anonymized at the time of examination, treated as blind (single-blind) to the operator, and randomly assigned a unique number. The sample will then be sent to a central laboratory to test for *SDC2* methylation. After study enrollment is complete, the methylation analysis results will be disclosed. The results and case report form (CRF) will be compared and analyzed to evaluate the sensitivity and specificity of the EarlyTect™-Colon Cancer test. Residual stool samples may be archived for further research. Clinical data and samples will be maintained in a manner that preserves the anonymity of all subjects. Specimens will be stored for 5 years in the on-site biorepository of the central laboratory for potential use in future research. A schematic diagram of the trial workflow is depicted in Fig. 1.

The *SDC2* methylation test will be performed according to the GT-CRC-UM-701 protocol (provided by the Sponsor) using the AB 7500 Fast Real-time PCR System (Thermo Fisher Scientific, Waltham, MA, USA). Briefly, 2 µg stool-derived genomic DNA will be chemically modified with sodium bisulfite using the EZ DNA Methylation-Gold kit (ZYMO Research, USA), according to the manufacturer’s instructions. For PCR analysis, *SDC2* and *COL2A1* control reactions will be performed twice in a single tube, as previously described [18, 19]. C_T_ values will be calculated using the software accompanying the AB 7500 Fast Real-Time PCR System. *SDC2* methylation will be considered detected if C_T_ is less than 40 cycles and not detected if C_T_ is not measurable [17]. To maximize clinical sensitivity, *SDC2* methylation will be considered positive if at least one of two PCR reactions has detectable methylated *SDC2*. Thus, samples will be categorized as positive if at least one of two reactions (1/2 algorithm) has detectable *SDC2* and considered negative if *SDC2* methylation is not measurable in both reactions. Our previous clinical study showed that the 95% limit of detection of the EarlyTect™-Colon Cancer assay was 34.5 pg, corresponding to approximately 20 diploid genome copies [19]. Reproducibility and repeatability of the assay were acceptable, within a coefficient of variation of 5%. A total of 23 potential interfering substances, selected because of their clinical and dietary uses in South Korea, were confirmed to have no effect on the test performance of EarlyTect™-Colon Cancer assay. The assay also exhibited no cross-reactivity, even when excess amounts (10^5^–10^6^ genome copies) of bacterial and viral DNA were tested.

Results from the EarlyTect™-Colon Cancer test will be compared against those obtained by colonoscopy and any corresponding diagnostic histopathology from clinically significant biopsied or subsequently excised lesions. Based on these results, participants will be divided into three groups: CRC (group 1), polyp (group 2), and negative (control). The following clinical data will be recorded for the participants: sex, age, colonoscopy results, and clinical stage (in the CRC case group).

Participant selection criteria are as follows:

*Inclusion Criteria*

1. No symptoms suggestive of CRC.
2. The individual provides written informed consent.
3. The individual is at least 60 years old when they receive a colonoscopy.
4. Criteria for high-risk CRC patients younger than 60 years old who will participate in the trial are as follows:

- At least one parent and/or sibling was diagnosed with colorectal cancer before age 55 years.
- One of his/her parents, brothers, and sisters was diagnosed with colorectal cancer after age 55 years.
- More than two of his/her parents, brothers, and sisters were diagnosed with colorectal cancer.
- Previous polypectomy for pathologically confirmed adenoma (at least 1 year ago).
- Occurrence of inflammatory bowel disease (diagnosed at least 15 years ago).

5. The individual provides more than 20 g of his/her stool for analysis.

*Exclusion Criteria*

1. Younger than 60 years and does not meet the aforementioned high-risk criteria.
2. Receiving or has received any type of therapy for CRC.
3. Less than 20 g of stool is provided for analysis.
4. Stool is considered inadequate for analysis (e.g., diarrhea or loose stool).

### Objective

This trial aims to validate the use of *SDC2* methylation in stool DNA for early detection of CRC in asymptomatic populations. The primary endpoint of this study is to evaluate the sensitivity and specificity of the EarlyTect™-Colon Cancer for detecting CRC.

### Sample size calculation

Because this is not a randomized controlled trial, a statistical calculation for comparative analysis was not considered. Instead, we have assumed that the prevalence of colon polyps in Korea is 25% based on our previous pilot study, in which we found an 80% sensitivity of EarlyTect™-Colon Cancer for detecting CRC and polyps in 585 patients [19]. Therefore, at least 476 participants are needed to identify polyps or cancers. In our previous study, we intentionally controlled the types of cases (i.e., 245 CRC, 44 adenomatous polyps, and 245 negative colonoscopy results). However, we will enroll more than two times the number of cases (n = 1000) in this trial and specifically include asymptomatic participants who will undergo colonoscopy. Assuming a drop-out rate of 10%, 1112 participants will be enrolled in this trial.

### Data collection and management

The authors will use an electronic CRF (Excel, Microsoft, USA) to record clinical data and information. Principal investigators or clinical research coordinators at each site will enter clinical data into the CRF. Approved researchers from the central data managing institution will manage the data and information. Only the principal investigator will be able to access the final trial dataset and analyze it. Personal information regarding the enrolled participants will remain anonymous for 5 years and deleted to protect confidentiality.

### Drop-out

A participant can be removed from the trial for the following reasons:

1. Submission of an unacceptable stool sample
2. Refusal to participate for any reason

### Statistical analysis

To calculate sensitivity and specificity, the test results will be denoted with dichotomous variables, namely “1” for methylation-positive individuals and “0” for methylation-negative individuals. Sensitivity and specificity for CRC detection, area under the curve (AUC) value, and 95% confidence interval (CI) will be calculated using receiver operating characteristics (ROC) analysis (SAS v.9.4). The cut-off value for C_T_ will be less than 40, and the following endpoints will be assessed:

- True positive (TP): *SDC2* methylation-positive in CRC patients
- True negative (TN): *SDC2* methylation-negative in the control (negative) subjects
- False-positive (FP): *SDC2* methylation-positive in the control subjects
- False-negative (FN): *SDC2* methylation-negative in CRC patients
- Sensitivity = 100 × TP/(TP + FN)
- Specificity = 100 × TN/(TN + FP)
- Negative predictive value (NPV) = TN/(TN + FN)
- Positive predictive value (PPV) = TP/(TP + FP)

### Safety evaluation and reporting of adverse effects

We anticipate no adverse events related to using the device in this trial.

### Data monitoring

A committee will be organized for trial supervision. All members of the committee will be certified according to Good Clinical Practices at each institution. The committee will monitor all processes, including data collection, records, and management, as well as advise and request the principal investigator to alter plans if needed.

### Protocol modification

If necessary, the protocol can be modified following agreement between the principal investigator and trial participants.

## Discussion

Using cancer-specific methylated DNA as a target for the early detection of cancer has several advantages, when compared with using known biomarkers [15]. (1) Unlike mRNA or protein, DNA is not easily degraded outside the body and maintains relatively high stability when isolated from body fluids for non-invasive diagnosis. (2) DNA can be amplified by PCR, which enables effective detection of minute quantities of methylated biomarker DNA in patient samples. (3) mRNA and protein markers require multiplexing to increase their accuracy in cancer diagnosis, whereas methylated biomarkers can diagnose cancer with a single gene and are not greatly affected by sample purity. (4) Biomarker levels can vary based on changes in emotional, physiological, or pathological factors that are unrelated to cancer; however, DNA methylation is highly robust in terms of detection because no such changes have been reported in vivo. (5) DNA methylation has a higher specificity for specific cancers than gene mutations, mRNA, or protein markers. (6) Unlike with other markers, early detection is possible with DNA methylation because methylation occurs in pre-cancerous lesions or early cancers.

Despite its accuracy, patients who undergo colonoscopy feel discomfort due to its invasiveness and the need for extensive bowel preparation. Therefore, a non-invasive and accurate novel screening technique can help make colonoscopy a selective procedure. As mentioned above, we previously demonstrated the diagnostic accuracy of the EarlyTect™-Colon Cancer test for detecting CRC in a case-controlled study [19]. EarlyTect™-Colon Cancer test exhibited high analytical sensitivity and specificity for *SDC2* methylation detection, as well as high reproducibility [19]. These results provided strong evidence that EarlyTect™-Colon Cancer test is optimized for analyzing stool-derived DNA. To further improve clinical sensitivity for detecting precancerous lesions, digital PCR or next generation sequencing techniques will be helpful to detect *SDC2* methylation in stool samples.

Our stool DNA-based test employs a single methylation marker *SDC2* for detecting CRC, whereas the multitarget stool-based DNA test called Cologuard (which is USA Food and Drug Administration-approved) assesses the presence of 2 methylation markers (*BMP3* and *NDRG4*) and *KRAS* mutations and includes FIT of stool samples. The *SDC2* methylation test has the same high sensitivity for detecting CRC as Cologuard [20]. Furthermore, the NPV of our test is comparable to that of Cologuard (99.9%) and FIT (99.7%), indicating that a negative methylation test result can provide similar information regarding the absence of CRC [18].

It has been reported that aberrant *SDC2* methylation occurs frequently in the early stages of neoplasia, is maintained in advanced CRC, and is unaffected by such factors as disease stage, age, sex, and race. These findings were observed in clinical studies comparing methylation of *SDC2* in stool DNA from patients with different stages of CRC, as well as from healthy subjects [17–19, 21, 22]. Several other studies have also reported tests for detecting CRC based on *SDC2* methylation in stool DNA [6, 21, 22]. Overall, these reports showed sensitivities of 77.4–81.1% and specificities of 88.2–98%, which are comparable to the values of our test.

Nevertheless, trials involving asymptomatic individuals are required to confirm whether the EarlyTect™-Colon Cancer device is a useful cancer-screening tool. Enrollment for this trial began on June 7, 2020 after registration was opened on ClinicalTrials.gov on March 11, 2020 (NCT04304131). This is the first prospective, multicenter trial to evaluate the clinical performance of EarlyTect™-Colon Cancer test in asymptomatic high-risk Korean populations. This test uses a real-time PCR assay to detect a single novel DNA methylation marker, methylated *SDC2*, in stool DNA. We were the first group to identify the usefulness of this biomarker, and we proprietarily developed the EarlyTect™-Colon Cancer test for early CRC detection. Some similar clinical trials have taken advantage of our discovery and used methylated *SDC2* as a biomarker in stool DNA tests for CRC detection; these tests exhibited very good performance, as expected. These similar trials utilizing methylated *SDC2* as a biomarker provide further evidence that *SDC2* methylation analysis is a useful noninvasive diagnostic approach for early detection of CRC, which may improve current diagnostic practices when used in conjunction with existing screening tools, such as FIT and colonoscopy [6, 21, 22].

We expect that this trial will provide results that will be beneficial for healthy people who want to avoid colonoscopy. Although the EarlyTect™-Colon Cancer test cannot match colonoscopy in terms of accuracy, it can still be used for early detection of CRC. This study protocol uses a prospective clinical study design to validate the effectiveness of *SDC2* methylation, a new biomarker for non-invasive early detection of CRC, in a relatively small population of high-risk individuals. This work should provide practical information for the design of a larger-scale prospective clinical study of an average-risk population to clarify the use of EarlyTect™-Colon Cancer test as a screening tool for CRC.

### Study status

A total of 231 patients were enrolled at the time of submission (November 2, 2020).

## Data Availability

Not applicable.

## Abbreviations

CRC: Colorectal cancer
*SDC2*: Syndecan-2
CRF: Case report form
TP: True positive
TN: True negative
FP: False positive
FN: False negative
FIT: Fecal immunochemical test
CT: Computed tomography
